# Identification of Novel Genes Involved in the Pathogenesis of an ACTH-Secreting Pituitary Carcinoma: A Case Report and Literature Review

**DOI:** 10.3389/fonc.2018.00510

**Published:** 2018-11-06

**Authors:** Fuyou Guo, Guoqing Wang, Fang Wang, DingKang Xu, Xianzhi Liu

**Affiliations:** Department of Neurosurgery, The First Affiliated Hospital of Zhengzhou University, Zhengzhou, China

**Keywords:** pituitary carcinoma, mutant genes, molecular mechanism, targeted therapy, ATRX, PTEN

## Abstract

Pituitary carcinomas (PCs) is considerable uncommon entities with a poor prognosis that represents only 0. 1–0.2% of all pituitary tumors. There are fewer than 150 reported cases up to now. In addition, the molecular pathogenesis leading to malignant pituitary transformation remain unclear due to the rarity of PCs. Here we present an uncommon case of ACTH-secreting PCs and explore the gene mutation following pituitary adenoma transformation. Our detailed clinical, histopathological and molecular detection data suggest that novel genes of ATRX and PTEN were implicated in the pathogenesis of PCs by searching Pubmed and the Web of Science databases as well as Cosmic databank. To the best of our knowledge, this is the first documented rare PCs patient with novel gene mutations that included ATRX and PTEN in addition to TP53. Present finding may therefore provide significant information for targeted therapy of PCs.

## Introduction

Pituitary carcinomas (PCs) constitute an extremely rare clinical entity that represents only 0.1–0.2% of all pituitary tumors, and there are fewer than 150 reported cases (1). PCs usually develop from progressive atypical pituitary adenomas and predominantly consist of hormone-generating tumors, defined by systemic metastases or the presence of disseminations to the cerebrospinal system. However, the molecular pathogenesis leading to malignant pituitary transformation is largely unidentified. A comprehensive understanding of the molecular mechanisms driving malignant pituitary progression would be beneficial for the treatment of pituitary carcinoma. Herein we described an uncommon PCs and the novel genes involved, providing useful evidence for future targeted therapy.

## Case description

**Figure 1:**
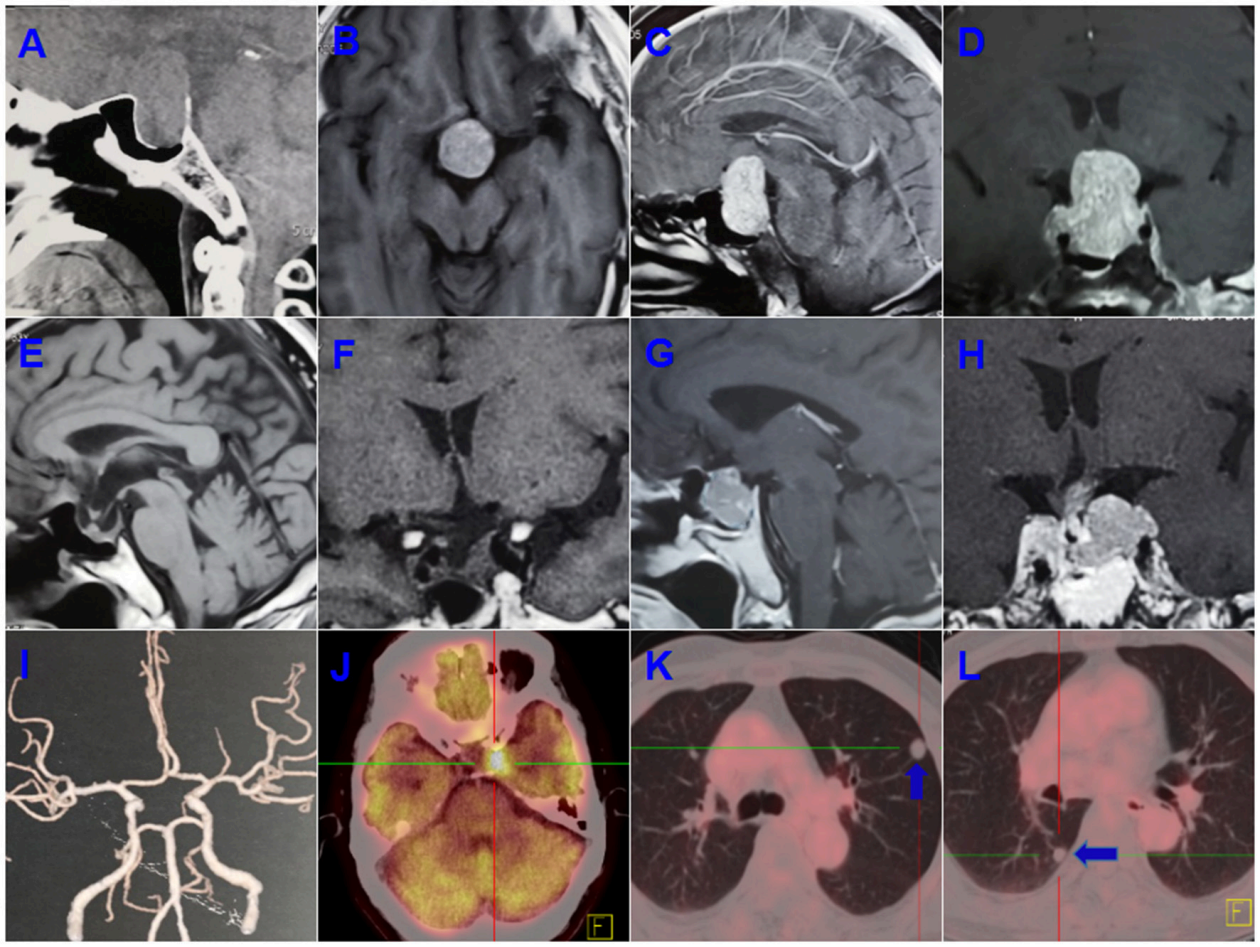
Preoperative computed tomographic (CT) scanning revealed a large mass lesion in the sellar region **(A)**. Magnetic resonance imaging (MRI) also demonstrated a large lesion located in the sellar region, with heterogeneous enhancement and invasion to both cavernous sinuses **(B–D)**. Subtotal resection was obtained after operation **(E,F)**. A recurrent tumor was involved in the saddle fossa and left cavernous sinus **(G,H)**. Computed tomographic angiography (CTA) ruled out a left posterior communicating artery aneurysm **(I)**. The PET-CT showed a residual intracranial tumor in the left cavernous sinus after a second operation **(J)**. Multiple metastatic lesions were found in the lung (**K,L**; blue arrow indicates the metastatic lesion).

A 55-years-old male presented with progressive deterioration of visual acuity and dizziness for 2 months. A preoperative computed tomographic (CT) scan revealed a large-mass lesion of the sellar region with extreme suprasellar extension (Figure 1A). Magnetic resonance imaging (MRI) demonstrated a large lesion located in the sellar region with heterogeneous enhancement and invasion to both cavernous sinuses (Figures 1B–D). The size of the tumor upon MRI was ~3.0 × 2.5 × 4.0 cm. Endocrinologic tests showed that the levels of adrenocorticotropic hormone (ACTH) were significantly elevated to 411.3 pg/ml (range, 7.2–63.3) at 8 a.m. and 352.1 pg/ml (4–32) at 4 p.m. The cortisol concentrations were 1,123.9 ng/ml (171–536) at 8 a.m. and 912.3 ng/ml (64–327) at 4 p.m. Other hormones, such as prolactin, growth hormone, free thyroxine (T3 and T4), and thyroid-stimulating hormone (TSH) were normal. The patient underwent an endonasal transsphenoidal surgery, and subtotal resection was obtained after the operation (Figures 1E–H). The postoperative ACTH levels dropped to 96.8 pg/ml at 8 a.m. and 78.3 pg/ml at 4 p.m., and the level of cortisol was reduced to 321.1 ng/ml at 8 a.m. and 165.2 ng/ml at 4 p.m. The residual tumor at the left cavernous sinus was subsequently treated with gamma knife surgery.

The ACTH and cortisol values remained stable during the 4 years of follow-up. However, 5 years after the first surgery, the patient was readmitted with a history of 20 days of left visual disturbance and 10 days of left eyelid ptosis. A MRI scan revealed a recurrent tumor in the sellar region and invasion of the left cavernous sinus. Computed tomographic angiography (CTA) was adopted to rule out an aneurysm of the left posterior communicating artery, and no aneurysm was found on CTA (Figure 1I). Hormonal evaluation showed slightly decreased levels of FT3 and FT4 (3.12 pmol/L [3.28–6.47] and 5.22 pmol/L [7.9–18.4], respectively). The ACTH levels were 41.3 pg/ml (7.0–61.1) at 8 a.m. and 38.6 pg/ml (3.5–30.55) at 4 p.m., and the levels of cortisol were 4.9 ug/dl (7–27) at 8 a.m. and 17.6 ug/dl (3.5–13.5) at 4 p.m. A second surgery was performed. The postoperative FT3, FT4, TSH, and cortisol values were significantly decreased compared with respective preoperative hormone levels. The patient was discharged under hormonal replacement therapy with euthyrox (25 ug/d) and cortisone acetate, and adjustment dosages were administrated based on subsequent endocrinologic tests. Postoperative histopathologic examination showed the presence of a PC. PET-CT was used for further evaluation and a residual intracranial tumor was observed in the left cavernous sinus (Figure 1J). Multiple metastatic lesions were also found in the lung (Figures 1K,L), and biopsy of these lesions revealed a metastatic neuroendocrine tumor.

**Figure 2:**
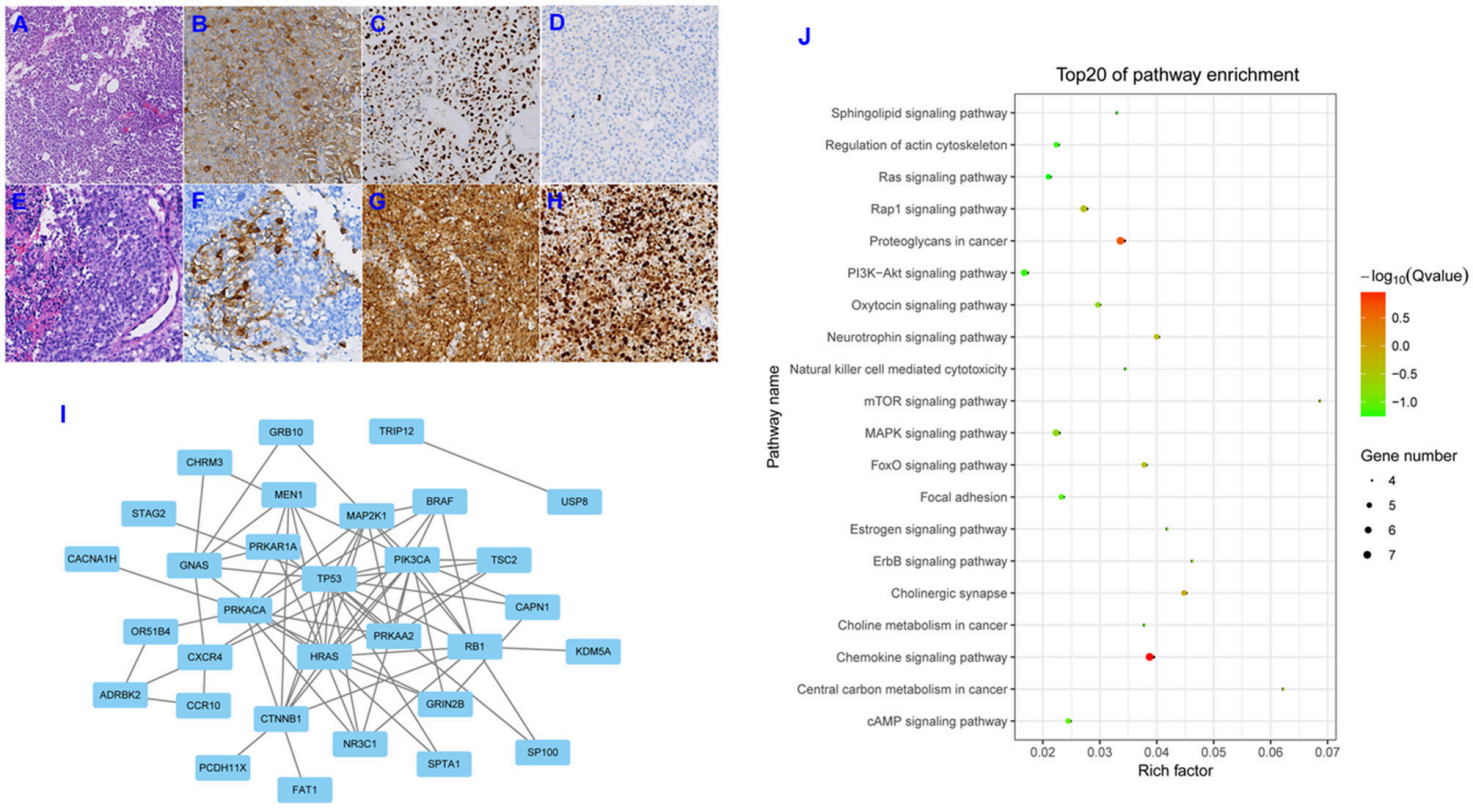
Hematoxylin and eosin (H&E) staining showed circular cells with uniform morphology and no heterotypic cells for the first operation specimen (**A**; original magnification, ×200). Immunohistochemical (IHC) staining revealed positive expression for ACTH and P-53 (**B,C**; original magnification, ×200), and Ki-67 expression was essentially negative (**D**; original magnification, ×200). H&E staining revealed excessive pleomorphic cells and frequent mitoses (**E**; original magnification, ×200). IHC staining was positive for the expression of ACTH (**F**; original magnification, ×200), and strongly positive staining for P53 and Ki-67 was observed (**G,H**; original magnification, ×200). Depiction of the protein-protein interaction (PPI) network for the 44 mutant genes involved in pituitary adenomas **(I)**. Depiction of the Kyoto Encyclopedia of Genes and Genomes (KEGG) pathways enriched for the 44 mutant genes **(J)**.

Initial postoperative histopathologic examination revealed a pituitary adenoma, and microscopic evaluation showed that the tumor consisted of circular cells of uniform morphology (Figure 2A). Immunohistochemical staining was positive for the expression of ACTH and P53 (Figures 2B,C), and Ki-67 expression was essentially negative (Figure 2D). A second postoperative histopathologic examination showed the presence of a PC. Hematoxylin and eosin staining also revealed the presence of excessive pleomorphic cells and frequent mitoses (Figure 2E). Immunohistochemical staining was positive for the expression of ACTH (Figure 2F), with strong positive staining for P53 and Ki-67 (Figures 2G,H); in fact, Ki-67 expression was up to 80%. In addition, we conducted a systematic review of the literature by searching Pubmed and Web of Science databases, and the Cosmic databank to ascertain all published studies on alterations in gene expression with respect to pituitary adenomas and pituitary carcinomas. Our literature search identified 44 mutant genes in pituitary adenomas. Their protein-protein interaction (PPI) network is shown in Figure 2I. Using these 44 genes, we found enrichment of several GO groups using the Kyoto Encyclopedia of Genes and Genomes (KEGG) database (Figure 2J). Among these GO groups were signaling pathways involving Ras, mTOR, MAPK, FoxO, ErbB, focal adhesion, and PI3K-Akt. However, we only uncovered limited novel gene expression changes and related clinical features from an isolated case report regarding PC (2–7) (see Table 1). In light of the exceeding rareness of PCs, molecular profiles of genes in the current rare case were derived by the Beijing Pangenomics Technology Co., Ltd. Only 3 gene mutations were found among 509 genes examined in the ACTH-producing PC in our study, including mutations in ATRX (alpha thalassemia/mental retardation syndrome X-linked) and P53. In addition, we uniquely identified the novel mutation in PTEN (Phosphatase and tension homolog deleted on chromosome 10) by comparison analysis with published data on PCs. The patient's residual tumor was well-controlled by temozolomide and general radiotherapy for 3 months, and there have been no new lesions or metastases.

**Table 1:** Summary of the literature regarding mutant genes involved in PCs cases.

**References**	**Sex**	**Age**	**PC type**	**Metastasis location**	**Mutation gene**	**Treatment**	**Follow-up**
Greenman et al. (2)	Female	37 y	Growth hormone secreting	Left neck lymph node	NA	Surgery+ Medicine	NA
Nose-Alberti et al. (3)	Female	22 y	ACTH secreting	Live	c-erbB-2	Surgery	Died after 4 months
Roncaroli et al. (4)	Male Female	55 y 53 y	FSH secreting FSH secreting	Skull base, nasal sinuses, and larynx, Vertebral bodies and ribs	HER-2/neu	Surgery Surgery + chemotherapy (cyclophosphamide, vincristine, dacarbazine)	Died after 2 years Stable 19 years
Scheithauer et al. (5)	Male	19 y	Thyrotropin secreting	Foramen magnum and cervical 2–3 levels	MEN1	Surgery + chemotherapy (octreotide)	18 years
Wei et al. (6)	Female	50 y	Non-functioning	Multiple intracranial metastases	miR-20a, miR-106b, miR-17-5p	Surgery + radiation + chemotherapy (Temozolomide)	Died after 8 months
Casar-Borota et al. (7)	Male Female	35 y 39 y	ACTH secreting	NA	ATRX DAXX	NA	NA
Present case	Male	60 y	ACTH secreting	Lung	PTEN, ATRX, P53	Surgery + radiation + chemotherapy (Temozolomide)	3-months follow-up remaining

*ACTH, adrenocorticotropichormone; ATRX, alpha thalassemia/mental retardation syndrome X-linked; DAXX, death-domain-associated protein; NA, none available; PTEN, phosphatase and tension homolog deleted on chromosome 10; y, years*.

## Discussion

PCs are considered uncommon entities and exhibit a poor prognosis due to their highly aggressive biology. Unlikely pituitary adenomas, PCs have received little attention, and the malignant transformation process from pituitary adenomas remains unclear. The present case is consistent with the criteria governing PCs as follows: (1) our patient experienced pituitary adenoma resection and subsequent adjuvant radiation as previous management; (2) a second postoperative histopathologic examination showed that the tumor was highly malignant, with Ki-67 expression up to 80%; and (3) multiple metastases into the lungs were observed by PET-CT, and biopsy of lung lesions revealed a metastatic neuroendocrine tumor. In addition, the most common category of PC was ACTH secreting (34.7%), prolactin secreting (23.6%), and null cell (15.3%). The latency period between the presentation of a sellar pituitary adenoma and the manifestation of metastases occupies a surprisingly wide range, from a few months to 18 years (median, 5 years). All accumulating evidence strongly supports the diagnosis of a PC, although it is an extremely rare tumor.

Temozolomide monotherapy is the first-line chemotherapy for pituitary carcinomas based on clinical practice guidelines from the European Society of Endocrinology (8). However, there is a paucity of randomized controlled trials for large-series studies; moreover, this drug is not sensitive and effective for all PCs. In fact, the estimated response rate to temozolomide is 58% in aggressive pituitary adenomas and 55% in PCs (9). Thus, it is necessary to elucidate the precise molecular mechanisms governing malignant transformation, which would then contribute to developing targeted therapy for PCs. Unfortunately, there have only been a few isolated mechanistic studies with respect to PCs described in the recent literature (Table 1). We exploited molecular profiles of PCs as they pertain to the pathogenesis of malignant transformation in our rare case; and conducted a comparative study from available data in the Cosmic databank (http://cancer.sanger.ac.uk/cosmic). Evidence showed that the gene mutation frequency for TP53 and HRAS were 33 and 14%, respectively, in PCs using the latest COSMIC databank. In addition, KEGG pathway enrichment analysis showed that the 44 mutant genes were enriched significantly within several signaling pathways, including Ras, mTOR, MAPK, FoxO, ErbB, focal adhesion, and PI3K-Akt. These enriched pathways provided insights into the molecular mechanisms underlying PC initiation and progression, and can therefore be useful in the development of new therapeutic strategies.

Our findings not only detected an uncommon P53 mutation from a total of 509 known genes, but also novel gene mutations in ATRX and PTEN unique to this PC. Mutations in ATRX and PTEN might, then, play vital roles in the malignant transformation of a pituitary adenoma into a PC. Most importantly, PTEN is a tumor suppressor gene that dominates the PTEN/AKT/PI3K pathway, prolonging progression-free survival to 11.4 months on pancreatic neuroendocrine tumors in a sunitinib group compared with 5.5 months in a placebo group (10). We assume that PTEN may be a crucial treatment target for PCs. Thus, we suggest that PTEN inhibitors, such as everolimus be used as an alternative chemotherapy for PCs once treatment failure occurs with temozolomide.

## Conclusions

To our knowledge, this is the first documented PCs patient with novel mutant genes, including ATRX and PTEN. The present findings will therefore contribute to the development of promising targeted therapy based on individual gene assay for uncommon PCs, although further studies of a larger cohort of PC patients are necessary to clarify the precise molecular mechanism(s) underlying the pathogenesis of PCs.

## Ethics statement

This study was carried out in accordance with the recommendations of frontiers in oncology. The protocol was approved by the medical ethical committee of Zhengzhou University. This patient gave written informed consent in accordance with the Declaration of Helsinki. I currently state that written informed consent was obtained from the participant for the publication of this case report.

## Author contributions

FG: study concept and design, acquisition of data and writing paper. GW: study concept, analysis and interpretation of data. FW: study concept and design, data collection. DX: analysis and interpretation of data. XL: critical revision of manuscript for intellectual content.

### Conflict of interest statement

The authors declare that the research was conducted in the absence of any commercial or financial relationships that could be construed as a potential conflict of interest.
